# Adult and Adolescent Disclosures of Child Sexual Abuse: A Comparative Analysis

**DOI:** 10.1177/08862605221088278

**Published:** 2022-04-28

**Authors:** Lucy McGill, Rosaleen McElvaney

**Affiliations:** 1 Rosaleen McElvaney, School of Nursing, Psychotherapy and Community Health, 8818 Dublin City University, Dublin, Ireland; 2 St Clare's Unit, Children's Health Ireland at Connolly, Blanchardstown, Dublin

**Keywords:** sexual abuse, adolescent victims, adult victims, sexual assault, reporting/disclosure

## Abstract

The recent attention focused on child sexual abuse (CSA) disclosure pathways has highlighted complex psychological processes that influence disclosure both for children and adults. Some authors have suggested that such processes may differ between children and adults yet few studies have examined distinct samples within the same study. This paper addresses this gap by exploring adolescent (*n* = 20) and adult (*n* = 10) experiences of disclosure of childhood sexual abuse. Interviews were conducted with both samples, using the same interview schedule and a comparative analysis was conducted of the key themes identified from a grounded theory analysis. Those themes that were found to be common to both samples included pressure cooker effect, telling would make it worse, and self-blame. Themes that were found to be more prevalent in the adolescent sample included police/court involvement, concern for other children, being asked, and peer influence. It is suggested that such potential differences reflect the changing social context over the past few decades which is characterised by increased awareness of sexual abuse as a crime and the risks of recidivism of offenders.

## Introduction

Child sexual abuse (CSA) can often go unnoticed, as physical evidence can be lacking (Paine & Hansen, 2002). Therefore, disclosing the abuse is the ‘single most significant’ means by which sexual abuse is discovered (Goodman-Brown et al., 2003). While there is a high prevalence of first time disclosures during adulthood (Collings et al., 2005; Collin-Vézina et al., 2015; Easton, 2013; Gagnier & Collin-Vézina, 2016; Hunter, 2011), these adult studies do not reflect societal changes and raised awareness of sexual abuse in recent years. Research with children and young people suggest that the increased focus on child abuse over the past few decades may account for findings that children are more likely to disclose abuse within two to three years following the experience of abuse than at any other time in childhood (up to 18 years) (McElvaney et al., 2021). Thus, it may be that children are beginning to disclose more promptly but more research is needed on both child and adult cohorts to examine this further. The current study investigates whether differences exist between adults’ and adolescents’ CSA disclosures in Ireland.

Disclosing CSA is seen as an ongoing, interactive, iterative process, with the person who discloses constantly evaluating how the disclosure is being received (Jensen et al., 2005; McElvaney et al., 2012; Staller & Nelson-Gardell, 2005). It is acknowledged that disclosure is challenging, with many intrapersonal, interpersonal, societal and cultural factors influencing the disclosure process across the lifespan (Alaggia et al., 2019). Nevertheless, it is often the first step towards stopping the abuse and accessing support and protection, both therapeutically and legally. Thus, delays in disclosure, particularly when these delays extend into adulthood, are a concern in society and improved understanding of disclosure processes is necessary to inform interventions. Early intervention is important not only to stop the abuse but also to limit the negative consequences of abuse (McElvaney, 2015a). The longer disclosure is delayed, the longer individuals are potentially living with the effects (e.g. serious physical and mental health problems such as trauma, anxiety, depression and in some cases, problems with addictions) without receiving any treatment, support or relief (Alaggia et al., 2019). Additionally, delayed disclosure poses the risk of further exploitation and further re-victimisation (Lalor & McElvaney, 2010).

It is estimated that between 55% and 70% of those who experience sexual abuse as a child delay disclosure until adulthood (London et al., 2008). Hébert et al. (2009) found that as few as one in five disclosed CSA during childhood. Lengths of delay vary but have been reported as up to 60 years (McElvaney, 2002). Alaggia et al.’s (2019) review of 15 studies of adults found that the mean age of disclosure for adult participants was between 40 and 50 years of age. Research has described adults as having more control over their disclosure as adults (Tener & Murphy, 2015), portraying adult CSA disclosures as a calculated and thoughtful process, culminating in a purposeful decision to disclose (Alaggia, 2004; Draucker & Martsolf, 2008). This view implies that adults are willingly telling or withholding abuse information and then making an intentional and deliberate disclosure (Del Castillo & Wright, 2009). However, as in young people, even during adulthood, disclosures can happen accidentally, resulting in the adult revealing or ‘vomiting’ the story (Draucker & Martsolf, 2008).

Experiences such as repressing memories, questioning whether or not the abuse happened, and feeling uncertainty or ambiguity about the accuracy of memories have been reported by adults who have experienced CSA (Crowley & Seery, 2001; Dorahy & Clearwater, 2012). Retrieved memories of abuse, while often provoking intense negative emotional experiences, can facilitate disclosures, which might help to provide meaning or control over the incident (Alaggia, 2004; Del Castillo & Wright, 2009). Studies have linked no disclosure with increased post-traumatic stress symptoms (Ullman, 2007); disclosing can be seen as a loss of control, which can in itself be distressing, particularly if they are not believed (Glover et al., 2010).

Studies have identified certain common themes when analysing factors that affected young people’s and adults’ decision to disclose CSA such as anticipated consequences, fear of negative consequences and their own emotions. Anticipated responses appear to be something that affects both adults and children in their decision to disclose and impacts further disclosures (Hunter, 2011; Morrison et al., 2018; Tener & Murphy, 2015). Specifically, many children experience fears about not being believed (McElvaney et al., 2014; Schönbucher et al., 2012); similar findings have been described with adults (Alaggia, 2004). Fear of negative consequences following disclosure is a common theme identified across age brackets, with young people often concerned about parental sanctions, ruining their reputation, threats, negative consequences for the perpetrator, violating family honour and being killed (Jensen et al., 2005; Münzer et al., 2016; Schönbucher et al., 2012; Shalhoub-Kevorkian, 2005). Both children and adults often fear hurting those they care about, concern for the feelings of others (Alaggia, 2004; Draucker & Martsolf, 2008; Hunter, 2011; McElvaney et al., 2014) and losing their post-abuse achievements such as their psychological balance, partner or children (Donalek, 2001). A further factor that has been found to influence both children and adults’ disclosure decisions has been the individual’s own emotional response to the abuse: guilt, shame and self-blame following abuse have been associated with delayed disclosure in both young people (Goodman-Brown et al., 2003) and adults following sexual abuse as a child (Alaggia, 2004; Dorahy & Clearwater, 2012; Draucker & Martsolf, 2008).

Studies of adults who were abused in childhood have tended to focus more on barriers to disclosure than on facilitators (Alaggia et al., 2019). In studies of children, factors facilitating disclosure include being exposed to conversations about sexual abuse (Malloy et al., 2013), being asked questions about their psychological wellbeing (LeMaigre et al., 2017), having access to a supportive adult (Morrison et al., 2018), concern for other children (McElvaney et al., 2014) and wanting to access support (Cossar et al., 2019).

Tener and Murphy (2015) advocated that in order to examine differences in adult and young people’s CSA disclosure process a qualitative approach might be best. Although there is much to be gained from quantitative methods (Ullman, 2007), sometimes specific details are not captured and the voice of the victims can be lost (London et al., 2008; McElvaney et al., 2012). Research has shown that open-ended questions allow children to provide more detailed and accurate accounts of their sexual abuse experience (Lamb et al., 2018). Therefore, qualitative research can provide a rich narrative of the disclosure process (McElvaney et al., 2014). While there appears to be some cross-over in relation to certain factors affecting CSA disclosure of young people and adults, Tener and Murphy (2015) state that adult CSA disclosure is something that is only partially understood and might actually differ greatly from that of children. Brennan and McElvaney (2020) caution that conflating findings from studies of adults and child survivors might result in important aspects and differences between the two groups being overlooked. While many studies have examined child and adult disclosure processes, very few studies have investigated these simultaneously and a lack of standardisation across studies makes comparisons extremely difficult (Lemaigre et al., 2017). Therefore, the current study bridges this gap in the literature by drawing on interviews with both adolescents and adults, so that factors affecting CSA disclosure can be directly compared between the two samples.

## Method

This study was part of a larger grounded theory study involving children, adolescents, parents and adults who experienced CSA in childhood (McElvaney, 2015b). The themes identified from the analysis of the larger study were used to conduct a secondary comparative content analysis of the adolescents’ and adults’ narratives.

### Participants

The sample consisted of young people (*n* = 20) (16 girls and 4 boys) between the ages of 12 and 19 (*M* = 15, *SD* = 1.85) and adults (*n* = 10, eight women and two men) ranging from 32 years of age to 63 years (*M* = 48, *SD* = 9.42), all of whom had experienced sexual abuse during childhood. Only two participants (one adult and one adolescent) were originally from outside of Ireland. Information on religiosity or ethnicity was not obtained. The types of abuse participants had experienced ranged from kissing to vaginal/anal penetrative and oral abuse. The young people interviewed were recruited through an assessment and therapy service for those impacted by childhood sexual abuse and most adult participants were recruited through a statutory counselling service for adults who have self-identified as having been sexually abused as children. One adult participant had a child also attending CSA therapy services and had been sexually abused themselves during their childhood. The adolescents had undergone a credibility assessment whereby professionals in a specialist assessment centre had offered an opinion that the young person had provided a credible account of CSA. This is standard practice in responding to sexual abuse in Ireland (Authors, 2015). Legal proceedings had not concluded in any of the cases included in this study at the time of interview. All participants were therefore already engaged with a therapy service and could be supported through the study. All relevant bodies provided signed assent/consent prior to data collection.

#### Adult Participants

This group consisted of eight women and two men. Age at onset of abuse ranged from 2 years to 11 years, with eight of the sample reporting abuse from the age of six to 12 years. Five of the adult sample had experienced abuse by more than one individual in separate abuse incidents. Eight individuals experienced intra-familial abuse (five of the abusers were relatives including father, brothers, mother’s partners and uncles), while two individuals experienced both intra-familial and extra-familial abuse. Extra-familial relationships included male adult family acquaintances, a teacher and a stranger. Participants described abuse experiences that ranged from a once off incident to over a period of years. The type of abuse experienced included penetrative abuse (vaginal or anal) for four of the sample and sexual touching for the remaining six. One of the latter was abused at a later stage where penetration was attempted.

The age at which they first disclosed ranged from 6 years to 40 years with five of these disclosures occurring during the childhood years up to age 11 and the remaining occurring between the ages of 19 and 40. Thus delays in disclosing ranged from no delay in one instance to 29 years. Those who disclosed during childhood had delays ranging from no delay to 8 years. The person to whom they first disclosed included boyfriend/girlfriend/wife in adulthood and mother/grandmother/peer in childhood.

#### Adolescent Participants

The young people interviewed in the study (*n* = 20) consisted of 16 girls and four boys. For the majority of children in the study, the abuse began before the age of 10, although delays in disclosure were evident and many of the children did not report the abuse until they were older than 10 years. The type of abuse experienced by the young people included genital and sexual fondling, kissing, oral sexual abuse, penetrative abuse, being undressed, being made to watch pornography, as well as vaginal and anal penetrative abuse. Sexual fondling was reported most frequently. In all cases, the abuser was known to the young person, and although the majority of cases were intrafamilial, some did report extra-familial abuse and two young people were abused both by somebody within and somebody outside their family. The extra-familial cases of abuse reported included abuse by a local man, a church sacristan and a family friend. Time to disclosure ranged from disclosing immediately to a delay of 9 years after the abuse began. Most of the young people first disclosed to a peer, with parent being the second most common choice of confidante (particularly among those who disclosed at a younger age).

### Data Collection and Analysis

The interview schedule used in the original study and the analytic process is described in (removed for blind review) and was informed by best practice protocols for interviewing children who may have experienced sexual abuse and based on Grounded Theory methodology (Charmaz, 2006; Strauss & Corbin, 1998). A number of open questions were used (e.g. ‘tell me about your experience of telling someone about what happened to you’; ‘what helped you tell?’, ‘what stopped you from telling earlier?’ and ‘what could we do to help children tell?’). The key themes of: signs of psychological distress, pressure cooker effect, concerns about other children, being asked, police, peer influence, telling would make it worse and self-blame were identified in the original study. This constituted the dataset for the current paper, which focuses on a comparative analysis of the presence of these themes in the adolescent dataset compared with the adult dataset. Interview transcripts and coding from the larger study were copied into a new database using the software programme NVivo (QSR, 2002) which was used for the data management and analysis. Each transcript was re-read and re-coded, using the themes noted above. Each author independently conducted a content analysis to establish the prevalence of each theme across adolescent and adult transcripts. Regular meeting were held to discuss the analysis in line with consensual qualitative research guidelines (Hill et al., 1997). Ethical approval was obtained from (University, community agencies).

## Findings

Both adolescent (denoted as ‘C’, followed by the relevant participant number) and adult (denoted as ‘A’, followed by the relevant participant number) participants expressed signs of psychological distress, evidence of the pressure cooker effect, a fear that telling would make things worse and self-blame as factors influencing their disclosure process (see Table 1). Four main factors differentiated adult and adolescent experiences, with more adolescents than adults identifying police/court involvement, concerns about other children, being asked and peer influence as factors influencing their disclosure experiences. Each of these themes are presented below with supporting quotes from participants to illustrate the themes (see Table 1).

**Table 1.** Prevalence of Themes Identified (Number and Percentage of Participants) in Both Samples.

Themes	Young People		Adults	
	Number (/20)	%	Number (/10)	%
Signs of psychological distress	8	40	2	20
Pressure cooker effect	13	65	3	30
Police/court involvement	13	65	2	20
Concern for other children	7	35	0	0
Being asked	11	55	1	10
Peer influence	16	80	1	10
Telling making it worse	17	85	5	50
Self-blame	6	30	4	40

### Signs of Psychological Distress

Both adolescents (*n =* 8) and adults (*n =* 2) described signs of emotional distress as a teenager resulting from the abuse experience:

I told him because I was really depressed …and I was close to him and he knew there was something wrong like he knew I wouldn’t be like that for nothing (C17)

I got fat when I was a teenager ‘cos he used to pay me money and I used to spend it on sweets mm and she took me to an obesity clinic once (A07)

Five of the adolescent girls interviewed were missing school or being disruptive at school and three of them were being rebellious at home as well. For example, C07 was displaying visible signs of distress that were being picked up on by those around her including arguing with her mother, disrespecting curfews, and hanging around with people with whom she wouldn’t normally have associated. C08 described regularly getting into trouble.

I was really getting in trouble for smoking in school and eh I was actually getting brought down to the office … they were wondering what was wrong with me why are you acting like this? And I broke down (C08).

C17 discussed how two of her friends told her parents that she was using drugs, an intervention which she appreciated in the long run ‘so they I think in their own way they did were trying to tell they didn’t know even at that time but they knew something’ (C17). Some individuals worried that they might be less likely to be believed because some of their distress had manifested itself in destructive or antisocial behaviours. For some of the adult participants, the psychological distress they experienced as a result of the abuse followed them into their adult years:

got to the stage I couldn’t sleep I used to wake myself up and I’d be awake for hours I saw him you know I’d see me Da coming in my sleep I couldn’t take myself out of the equation so what I was really beginning to suffer was exhaustion and it took me about 2 months to realise that he couldn’t hurt me anymore d’ya know that I’m an adult now it was I went back there d’ya know what I mean (A03)

### Pressure Cooker Effect

Both the young people (*n =* 13) and adults (*n =* 3) interviewed in the present study showed signs of the pressure cooker effect (an emotional build-up of pressure that leads to a disclosure; McElvaney et al., 2012) in their disclosure stories. The emotional build-up partially results from the energy needed to keep the secret and withholding the information about the abuse, which was something that both young people and adults carried with them for many years. One young person described how they tried so hard to tell somebody for about 2 years ‘aw it was unreal’ (C09), while according to one adult, ‘I carried that for over 30 years, you know’ (A04). Even though they wanted to tell somebody about the abuse, many experienced an internal battle, bouncing between wanting to tell but not being able to bring themselves to do it. For many, the need to tell became too much and resulted in psychological distress: ‘I never actually planned to turn round and tell somebody … but I suppose everything just builds up and then finally just comes out’ (C09). One woman spoke of how difficult it was to listen to her mother talk about a child who has been abused and how this prompted her to disclose:

she went on about this little boy being abused and dya know ‘dreadful dreadful dreadful’ and you know it was all building up inside of me and just out of nowhere, well not out of nowhere, I just said to her (A08)

### Telling Would Make It Worse

Both young people (*n =* 17) and adults (*n* = 5) expressed a fear that if they told somebody about the abuse, things would get worse, either for them, for their family or for the abuser. Many were worried they would upset their parents:

I didn’t want to tell me Ma about what really happened and worry her… I just didn’t want her to worry coz she had so much on her plate with me like cutting myself and trying to get better an I just didn’t want to worry her any more (C02)

there were so many things going on for me mother I think I didn’t want to cause any more upset (A07)

Others were worried about the potential consequences for their family. These included fears that their family might separate, that the abuser would be taken away and the family would struggle to survive financially.

I goes oh no I don’t want to tell my Mam she she’ll just be upset and I don’t want her to live on her own and how we’ll get food and everything so I was worried how I was going to how if I did tell em how would we cope with the family how would we live and would we have the money will we be able to? (C13)

Some of the young people had received death threats from their perpetrator and the fear that those threats might be enacted upon also prevented disclosure: ‘…he said you better not tell your ma or da or else I’m gonna I’ll eh kill you’ (C11); ‘he actually told me… if I told anyone I was gonna die so’ (CO3).

For two adult participants, their attempt at disclosing the sexual abuse was met with physical violence: ‘so me Mam em she hurt us over it cos she said no it never happened you know’ (A11); ‘I remember one time trying to tell me Grandmother and she she gave me a slap across the face and sent me down to the shop for eggs so then I didn’t tell again’ (A03). The temptation to leave things as they were and not make things worse or cause trouble was evident ‘Like I thought it would’ve if nobody knew it wouldn’t cause any pain for anybody only myself’ (C02). One of the adult participants also commented that their reason for not having disclosed at a younger age was a fear that there would be trouble if they had told, even though looking back they felt that probably telling their mother would have made things better:

I just didn’t want to cause trouble…I just got this thing in my head that I’d invented it, didn’t help, he’d get taken away and there’d be a load of trouble I just never said anything …I think he’d have probably have had a good telling off from me mother yeah when I look back it, probably wouldn’t a gone, wouldn’t gone ought like that really, she’d a probably just gone mad then she’d a been right upset … I’d say it probably woulda stopped cos I’d know it would be safe for then to say if you do it anymore I’m going to tell me mum again kinda thing (A07)

### Self-Blame

Self-blame was a prominent theme throughout both young people’s (*n =* 6) and adults’ (*n =* 4) narratives. Sometimes individuals blamed themselves for the abuse in the absence of other explanations. Pretending it wasn’t happening, inaction or not fighting back also led young people to believe that the abuse was their fault. One adult did not tell as she felt responsible for being near her alleged perpetrator’s place of work ‘and I felt I was to blame because I was hanging around you know this place where this man was working you know … so the best then would be not to tell’ (A08).

Self-blame was not always an immediate response, in some cases it developed over the course of a number of years, often without being challenged:

it wasn’t even about the threat anymore, it was about, because I thought it was my fault… (C17).

I mean you say to yourself God why did I not eh run out of the room when he opened the door ….why could I not say to me Ma ‘now Ma I don’t want this man here he’s eh messing around with me’ ….I really I musta been really thick….it’s always that shame that you feel God I coulda stopped that you know? (A09).

For others, time and perspective helped them to realise that they were not to blame:

I have came to terms with the fact that ok it wasn’t my fault… I woulda been ashamed of is the fact that I had let him because I had no choice….you know em it’s my fault so what’s the point in saying anything?….I think when you’re told then ok yeah he came into my room so that’s where your brain your brain is starting to say ‘well I wasn’t I didn’t go into his room’ and start making you realise then ‘oh God maybe is that my fault’?… you know that he was the one that approached my room I didn’t approach his room (A11)

Keeping the abuse a secret appeared to deprive participants of the opportunity for their own beliefs about self-blame to be challenged by others:

after going through a couple of years of actually thinking that it was your fault then I didn’t tell anyone because it was my fault. …when you’re like about 12 and you don’t have anybody to talk to ... you do start believing that it was your fault so you don’t tell anybody …why would I tell somebody? Why would I tell somebody something that it was my fault that I did it? (C17)

it’s just I have the understanding that I obviously allowed it to happen why wasn’t I strong enough? Why didn’t I shout? Like I mean there was seven of us there … in one bedroom …so he just had to creep in when everybody was sleeping you know … why did I allow that to happen you know? (A06)

### Police/Court Involvement

The majority of the young people involved in the study had contact with the police and criminal justice system (*n* = 13), while only two of the adults had such involvement as a child. For some, knowledge that police would be involved acted as a deterrent: ‘I didn’t want to tell…when they said that I could go to court with it but he’d be put in jail I did not want that…I didn’t want the feeling that I put him in jail like that’s just a terrible feeling for me’ (C13); ‘Cos you know gards^1^ are rats sometimes and they can muck about everything so em that’s why I don’t like going to the gards’ (C18); ‘where I come from, the gards, they don’t believe children…’ (C03); ‘Oh God is it worth all this?…didn’t really want the police brought in an everything…I was thinking God I shoulda kept my mouth shut you know that part all the police and all the trouble’ (C04). One young person disclosed to her mother a year after the assault as she thought that by then she would not have to make a statement, however she had no choice in the matter and was brought to the police: ‘Like there’d be absolutely no proof then so what would be the point?… …so it’s hard enough living in the same area as him than living in the same area as him and having gone to the guards’ (C07). However, C10 found dealing with the Gardaí^1^ easier than expected:

it was actually easier saying it to the Garda^1^’s face with my Mom beside me because like I didn’t have to say I didn’t have to come up with everything myself. The garda knew what kind of questions to ask me and I was able to answer without you know. She was able to explain to my Mom and stuff (C10).

Young people noted that the length of time that the legal proceedings were taking made it difficult for them to deal with the abuse and move on with their lives: ‘that’s what me Ma was saying, it could take years. An I was like but I’m trying to get over it how can I get over it if they’re gonna keep bringing it back up?’ (C20). When the outcome of the Garda investigation resulted in the Director of Public Prosecution (DPP) not proceeding with the prosecution, young people described feeling that they had not been believed or that it had all been a waste. ‘I couldn’t believe like I felt like everything was a waste…Everything seemed to be dragged out so much for nothing’ (C09).

Only two adults interviewed had reported their experience of sexual abuse to the police. A08 described how ‘all hell kinda broke loose, the police were called eh police car came down and took us away’ when she disclosed what had happened to her at age 6. A09 was first sexually abused at age 9 and experienced both medical and police intervention at that time. Both adults stated clearly that the police intervention inhibited them from further disclosing when they were abused again (both at age 11): ‘getting into trouble because I thought the police and the shame you know bringing all that on the family you know having to go to the police was a terrible thing you know’ (A08).

### Concern for Other Children

Concern for other children was a theme that was raised among the young people interviewed (*n* = 7) but did not feature in adults’ stories of disclosure. Consideration of the risk to other children acted as a motivator for disclosing the abuse:

(the children) were me main worry. I thought like he could do that to me and I can’t tell anybody.he can do it to them and they won’t tell anybody. And if I hadn’t told and a few years later (names of sibling) turned around and ‘well he done that to me a year after what he done that to you’ I woulda never forgiven meself I really woulda just lost it altogether (C09)

if anything happened them children I would have been devastated…and I just wasn’t gonna let that happen (C14)

then I just told them cos of my little brother and sister… I kept thinking of my little brother and sister and what would happen to them like” (C20).

Even though concern was expressed for other children, this did not always make the disclosure decision less complex. One young person spoke of wanting to protect her younger siblings but the conflict between wanting to protect them and an appreciation how upset they would be if their father had to move out of the family home was a cause of great distress for her. Following her disclosure, this man was removed from the family home and the children were indeed very distressed. This led to her retracting her allegation so that he could move back into the home, although the truth was subsequently revealed.

### Being Asked

Many of the young people in the study (*n* =11) described being asked questions either by parents, professionals or friends, that led to a disclosure: ‘then me ma asked me did ever anything happen to me and I just told’ (C12). While some young people were asked explicitly whether they had experienced abuse, others were asked more generally if there was something upsetting them ‘Ma was just asking me what was wrong then and just I says he tried it on with me’ (C02). For some young people, the abuse was observed by others or people had picked up on sexualised behaviour or strange cues in their social interactions: ‘She (friend) kept on asking me “Are you ok?”…“what is he doing to you?”...she just kind of knew I dunno how but she knew’ (C03). One teenager described how her mother knew there was something up by the look on her face when they were watching a television programme about abuse. One teenager, when asked if they would have disclosed had they not been asked, responded: ‘I don’t think I would’ve said yeah but I think I would have made it quite obvious that’s what woulda happened’ (C17). One young person described how it would have been easier to disclose if they had been asked more questions:

if my Mam told me what I had said, if someone had said like after I had said it that this is what you said…instead of going oh yeah ok… It’s so much easier to say it with the like someone’s asking you stuff instead of you having to think of what’ll I say first” (C10).

Only one adult described an experience that fitted with this theme. When asked to summarise what had really helped her tell, she responded ‘I think it’s the direct question really’:

There were times I’d cry or you know I’d waken up from nightmares and he (boyfriend) thought that it was him you know like if you’re in. It’d be like flashbacks… he wanted to know what was wrong you know. Was it him? or what was happening? and so I told him (A11).

Being asked was sometimes the trigger for disclosing, whereas for others it was just part of the disclosure process, both initially and for subsequent disclosures. For some, the first time being asked led to denial. One young boy’s sister asked him if his cousin had ever touched him after finding the cousin behaving inappropriately with him ‘…and I kinda started crying, said “no, no, no” and walked out, ran out of the room and went back onto the road’ (C08); however, he said this did provide indirect encouragement to disclose later.

Whether a disclosure followed being asked also depended on who asked them, sometimes it was parents, counsellors, boyfriend etc. One teenager described how she would have told her mother if she asked, but would not have told her teacher. Also, as one young person pointed out, being asked is not always enough as ‘it depends on whether the person’s ready to tell or not’ (C08).

### Peer Influence

Many of the young people in the study (*n* =16) remarked that they first confided in a peer. Peers were seen for many as being on their level, with one young person feeling that she could turn to her best friend over her family ‘they were the best person for me before I never really thought about telling anybody else because em I suppose I didn’t wanna upset the way things were in the family’ (C14). For some, peers helped them to realise the severity and gravity of the situation: ‘I never believed it for a long time until I actually told me friends and they were like listen you know what I mean this is major’ (C14).

Many young people discussed how it was their peers who eventually persuaded them to tell an adult. One young person’s friend responded: ‘what if he goes and does it again like why don’t you? You just be the one to deal with it now’ (C07) while another young person noted: ‘Look what could happen to your sisters and when I thought of that ...I was like no I’ve got to tell I don’t want it to happen to them I don’t want their lives ruined’ (C13). This young person’s friend persuaded her that telling her mother would be the right thing to do.

For the majority of the adult participants, confiding in a friend was rarely mentioned. As one adult noted,

I didn’t have many friends because I wasn’t that close to very many friends as I am now … but I wouldn’t have discussed anything like that with my friends no not at that age at all it was just you didn’t do it. You were afraid you were kept it to yourself (A02).

Only one adult participant first confided in a friend (other than a boyfriend/partner). She was an adolescent at the time and the abuse happened when she was seven to nine years old.

I remember walking to school one morning…I just said to her ‘oh yeah my granddad used to touch me’. And she were like ‘did he?’ and that were it. Then we started talking about something else and that were that (A07).

## Discussion

This paper compared the CSA disclosure experiences of adolescents and adults interviewed within the same study with a view to comparing factors influencing disclosure. Some themes featured in both adolescent and adult narratives – signs of psychological distress, the pressure cooker effect, telling would make it worse and self-blame – while the themes police/court involvement, concerns for other children, being asked and peer influence were more evident in the adolescent interviews than in the adult interviews. While it is important to acknowledge that there appear to be universal experiences that are likely (though not inevitable) following sexual abuse, there may be distinctive features that differentiate adolescents’ experiences from those of adults. This may be due in part to the more protracted impact of keeping the abuse a secret or it may be influenced by developmental factors and wider system issues. Factors that appeared to be more prevalent in young people’s narratives are discussed with a view to gaining a better understanding of the distinctive needs of adolescents and adults in facilitating disclosure.

Many of the young people interviewed had contact with the legal system in relation to their abuse. Only two adult participants in the current study had had contact with police in relation to the sexual abuse they experienced. This discrepancy may be explained by four main factors. First, most of the adults interviewed had not disclosed to anyone prior to adulthood. This is consistent with other community studies of adults who experienced sexual abuse in childhood (Alaggia et al., 2019; McElvaney, 2015a). Second, changes in national policy in Ireland over the past few decades have resulted in legislation mandating professionals to report child abuse to child protection agencies and the police (Government of Ireland, 2015). Adolescents in this study were recruited through a state agency where adherence to national policies of reporting abuse to the police was a requirement. For the adults in this study, no such requirement would have existed in their childhood. Many studies have found a low proportion of sexual abuse complaints filed with legal authorities as an adolescent or child in adult samples, both in Ireland and interntionally (Gagnier & Collin-Vézina, 2016; Hunter, 2011; McGee et al., 2002). Tener and Murphy (2015) suggest that future research should investigate the process of disclosing to different recipients such as legal professionals.

Third, adults have more autonomy in deciding whether to come forward and report their experiences of abuse; adolescents are often brought to agencies rather than reaching out of their own accord. Of note, in a study of high school adolescents in Sweden who reported having experienced sexual abuse (1505 girls and 457 boys), only 6.8% of them had reported the abuse to authorities (Priebe & Svedin, 2008). Many of the adolescents in the current study spoke of their lack of agency in relation to police involvement. In the case of both adults in this study who had reported to the police, they exercised their agency by not disclosing later abuse following unpleasant experiences with the police at a younger age. In a national survey in Ireland (McGee et al., 2002), only 8% of adults who disclosed sexual abuse as a child had reported this to police. Legislation directly addressing sexual offending and the introduction of specialist training for police and police interviewing of children and vulnerable adults has brought some improvements in how police in Ireland respond to such individuals; recent studies have highlighted positive experiences by adults of police investigations, despite finding the overall legal process traumatic (Brown et al., 2019). Increased engagement with the legal system for victims of sexual violence will provide opportunities for further research in this area. Finally, some of the young people in this study were supported by friends to report their experience of abuse to authorities. This in itself is evidence of increased awareness among young people of sexual abuse as a crime. Increased media attention in Ireland, the introduction in the early 90s of child protection programmes in schools, child protection policy development followed by mandatory reporting legislation, the development of dedicated interview suites in police stationsand several high profile state investigations into sexual abuse in Ireland in recent decades have clearly had an impact on the awareness of young people of the criminal nature of the offence, the risk of recidivism and the need to report experiences of sexual abuse to authorities.

This awareness of sexual abuse was also evident in young people’s concerns for other children, which for many appeared to facilitate their disclosure. Previous research has found that children are often conflicted as to how to best protect others and whether that involves non-disclosure (McElvaney et al., 2014) or disclosure (Shalhoub-Kevorkian, 2005), which highlights young people’s awareness of the impact of their disclosure on others. Many adults who have not disclosed in childhood engage in avoidance as a coping strategy to manage the psychological distress associated with their memories of abuse. Alaggia (2004) noted that geographical distance between adult survivors and the person who abused them may explain why adults often do not include self-protection as a reason for disclosure. This geographical distance may also protect adults from concerns about risks to other children.

A further discrepancy between adults’ and adolescents’ experiences in this study relates to ‘being asked’, a theme commonly featured in young people’s narratives but only mentioned by one adult. Being prompted or asked directly was cited as the most common reason for disclosure in a review of 13 studies examining disclosures of CSA as children, conducted by Lemaigre et al. (2017). Disclosure is a dyadic and interactive process and both younger and older children appear to be significantly more likely to disclose in a setting that provides prompts or questions about sexual abuse, such as an interview (Kogan, 2004; McElvaney et al., 2014; Schaeffer et al., 2011; Ungar et al., 2009). Over half of the children in Malloy et al.’s (2013) study attributed having disclosed to external factors, such as a presentation in school or a television programme. For children, having the appropriate information about sexual abuse seems to be important (Lemaigre et al., 2017), but programmes focussing on providing opportunities for children to tell, including directly asking children if they had been sexually abused, might be of benefit (Brennan & McElvaney, 2020). As well as advocating for educational programmes and information for child and adult recipients of CSA disclosures, Lemaigre et al. (2017) concluded that ‘the optimal condition for a disclosure is for an individual to directly ask the child about their experiences and that this individual provides active listening and support’ (p. 49).

Adults too, however, need to be provided with opportunities for disclosure. The Sexual Abuse and Violence in Ireland (SAVI; McGee et al., 2002) report showed that of the 1466 participants, 47% of those who had inappropriate sexual experiences as a child or adolescent had not told anybody about that experience prior to taking part in the survey. When asked in follow up telephone calls why they had not disclosed prior to the survey, a common theme identified was that they had not been asked. External prompts such as media stories (including those concerning legal sexual abuse cases or where celebrities disclose their history of abuse or victimisation) can also aid adult CSA disclosures (Kennedy, 2011). Mainstream attention to such topics can help individuals to feel approved, legitimated and a part of society (Tener & Murphy, 2015). Recent attention to the relationship between adverse childhood experience and later physical and mental health difficulties has highlighted the need to include questions in routine screening interviews with adults about childhood experiences such as CSA and to sensitively respond in a trauma informed manner that could significantly help those adults who are in need of psychological support related to their childhood experiences of abuse.

Few adults in the current study had disclosed to peers about their experiences of sexual abuse, either in childhood or in adulthood, other than to a partner, while many of the young people had confided in a friend. This finding in relation to adults’ non-disclosure to peers appears to contrast with other studies that have found that adults often did disclose to a peer (Denov, 2003; Draucker & Martsolf, 2008; Hunter, 2011; Isely et al., 2008; Jonzon & Lindblad, 2004). Choice of confidant can vary by age; it has been suggested that young children are more likely to disclose to a parent, adults to a peer and older adults to a professional (Roesler & Wind, 1994). However, peers appear to play a supportive role in the disclosure process in studies across age ranges with young people (e.g. Crisma et al., 2004; Priebe & Svedin, 2008) as well as with adults (e.g. Draucker & Martsolf, 2008; Hunter, 2011; Isely et al., 2008).

One consequence of CSA documented in previous research is difficulties forming trusting relationships following the abuse (Isely et al., 2008; Tener & Murphy, 2015). Thus, impact on the individual’s relational capacity (McElvaney et al., 2019) may impact on their access to a trusted confidante, which in turn may impact the likelihood of disclosure and subsequent access to support. However, as almost half of the adults in the current study did confide in a partner, this may reflect developmental differences between adolescents and adults. Peer relationships change over the lifespan with adolescence being a peak period of closeness to one’s peers. Roesler and Wind (1994) in their sample of adult females identified different patterns in disclosure across the lifespan: disclosing in childhood to a parent, in adulthood to a friend, partner or other family member and in later adulthood to a therapist or professional.

### Strengths and Limitations

Although previous authors have highlighted developmental factors in disclosure processes (Lemaigre et al., 2017; London et al., 2008; Paine & Hansen, 2002), this is the only study of which the authors are aware that drew on both an adolescent and adult sample in exploring disclosure processes and the factors that influence these processes. In addition, by focussing on adolescents (aged 13–18) and not younger children, two samples were captured where, it could be argued, participants had comparable cognitive ability to reflect on and offer an analysis of their experiences of sexual abuse. The findings, therefore, are significant in highlighting both similarities and discrepancies between these age cohorts, capturing changes over time in one cultural context, Ireland. Nevertheless, even for the populations’ demographics in Ireland, this is quite a homogenous sample. Males are under-represented in both the adolescent and adult samples. Only one young person and one adult were originally from another country. None of the sample represented populations of learning disabled or ethnic minority groups.

The potential for hindsight bias (Lemaigre et al., 2017) for both samples must be acknowledged, and many of the adults were reflecting on experiences they had four to six decades earlier, thus difficulties with recall are inevitable and age-influenced re-interpretations of events are likely (Malloy et al., 2013). Further research on disclosure across the lifespan is needed (Alaggia et al., 2019). Although the total sample size of 30 is robust for a qualitative study, comparative analysis is limited with such small sub-samples of 20 adolescents and 10 adults. For example, information relating to participants’ degree of religiosity was not collected in the current sample. Themes relevant to religiosity or ethnicity did not emerge in the inductive analysis of the larger study, possibly due to the homogeneity of the sample. A larger and more diverse sample could enable an analysis of the comparative influence of religiosity and ethnicity over time by comparing adolescents and adults from different ethnic and religious groups. Similarly, differential impact on disclosure based on the relationship between the child and the abuser could be explored in a larger more diverse sample. Most of the individuals in this study had experienced intrafamilial abuse. Recent research in Ireland exploring the impact of CSA disclosure on siblng relationships (Crabtree et al., 2021; McElvaney et al., 2021) highlights challenges facing adult siblings that would likely differ from family members of adolescents who have experienced sexual abuse. Finally, all of the participants in this study were engaged with therapy services. A community sample of adults and adolescents may reveal different dynamics that impact on disclosure.

## Conclusion

Tener and Murphy (2015) acknowledge that disclosure processes are affected by ever-changing social norms, so that the context in which disclosure processes unfold can change drastically from generation to generation. The current study, by capturing both adolescents’ and adults’ experiences of sexual abuse disclosures within one culture (Ireland), spanning several decades, highlights how societal changes such as raised awareness of sexual abuse as a crime, the risks associated with not reporting sexual abuse to authorities, and possibly changes in peer relationships impact on disclosure processes. The question of whether any real change in disclosure pathways has taken place in the past few decades and the potential impact of societal awareness and prevention programmes would need to be investigated in a much larger sample using inferential analyses. The diversity of experiences in terms of sexual abuse and the factors influencing disclosure raises questions as to whether comparisons between age cohorts are meaningful or worthy of investigation. McElvaney et al. (2019) suggest that the search for patterns in the factors that influence disclosure denies the reality of the unique experience of each individual and their psychosocial context.

## Note

1. “gards”, “Gardaí”, “garda” all refer to members of An Garda Síochána, the police force in Ireland
